# Mechanistic insights of Li^+^ diffusion within doped LiFePO_4_ from Muon Spectroscopy

**DOI:** 10.1038/s41598-018-22435-1

**Published:** 2018-03-07

**Authors:** Ian D. Johnson, Thomas E. Ashton, Ekaterina Blagovidova, Glen J. Smales, Mechthild Lübke, Peter J. Baker, Serena A. Corr, Jawwad A. Darr

**Affiliations:** 1 Department of Chemistry, University College London, 20 Gordon Street, London, WC1H 0AJ UK; 2 Diamond Light Source Ltd, Diamond House, Harwell Science and Innovation Campus, Didcot, Oxfordshire OX11 0DE UK; 3 ISIS Pulsed Neutron and Muon Source, STFC Rutherford Appleton Laboratory, Harwell Science and Innovation Campus, Didcot, Oxfordshire OX11 0QX UK; 4 School of Chemistry, University of Glasgow, Glasgow, G12 8QQ UK

## Abstract

The Li^+^ ion diffusion characteristics of V- and Nb-doped LiFePO_4_ were examined with respect to undoped LiFePO_4_ using muon spectroscopy (µSR) as a local probe. As little difference in diffusion coefficient between the pure and doped samples was observed, offering *D*_Li_ values in the range 1.8–2.3 × 10^−10^ cm^2^ s^−1^, this implied the improvement in electrochemical performance observed within doped LiFePO_4_ was not a result of increased local Li^+^ diffusion. This unexpected observation was made possible with the µSR technique, which can measure Li^+^ self-diffusion within LiFePO_4_, and therefore negated the effect of the LiFePO_4_ two-phase delithiation mechanism, which has previously prevented accurate Li^+^ diffusion comparison between the doped and undoped materials. Therefore, the authors suggest that µSR is an excellent technique for analysing materials on a local scale to elucidate the effects of dopants on solid-state diffusion behaviour.

## Introduction

Spin-polarised muons can be used as a local probe to investigate the solid-state diffusion behaviour of Li-ion battery materials. The diffusive processes within these materials are not always well understood and muons can provide detailed insight into the Li^+^ diffusion mechanisms^1–3^. The sensitivity of the embedded muon to local magnetism (through the time-evolution of its spin polarisation) has been utilised to investigate many properties of solid state materials, such as hydrogen diffusion, magnetism and radical chemistry^1–3^. Similarly, Li^+^ diffusion within a sample, perturbs embedded muons and the Li^+^ diffusion coefficient can be extracted from analysis of this perturbation. This technique has successfully determined the Li-ion diffusion coefficients in LiCoO_2_^4^, bulk LiFePO_4_^5–7^, nano-LiFePO_4_^8^, and the Li_6.5_Al_0.25_La_2.92_Zr_2_O_12_ solid-state electrolyte material^9^. Indeed, µSR has successfully determined consistent experimental diffusion coefficients of Li^+^ in pure LiFePO_4_ in the range 10^−10^ to 10^−9^ m^2^ s^−1^, in good agreement with theoretical studies, but to the authors’ knowledge has not been extended to V- or Nb-doped LiFePO_4_ systems previously^5–8^.

LiFePO_4_ (and doped variants) have been extensively investigated as a cathode material for Li-ion batteries, as it offers a more sustainable alternative to cobalt-based cathodes such as LiCoO_2_ and LiNi_*x*_Mn_*y*_Co_*z*_O_2_^10^. Historically, LiFePO_4_ initially suffered from poor Li insertion/extraction kinetics^11^; efforts to nanosize^12,13^, carbon-coat^14–16^, and dope the material have often improved the attainable storage capacity, particularly at high charge/discharge rates. In particular, aliovalent doping of LiFePO_4_ with transition metal ions such as V^3+^ and Nb^5+^ has been a successful strategy for improving the resulting electrochemical performance^17,18^, although there is a lack of consensus on the precise reasons for this. Many authors have observed distortions of the unit cell^19–22^, and widening of the 1D diffusion channels in the material, which was suggested as a mechanism for lowering the activation energy for Li-ion diffusion. There is also some argument as to whether the dopants create defects in the material (such as Li vacancies) that boost diffusion^20^. Furthermore, the dopant may alter the electronic conductivity and consequently improve performance^18^. The subtle effects of dopants on atomic structure can be difficult to observe in great detail using standard lab based analytical techniques, and therefore, alternative methods must be sought to fully understand the effects of doping on electrode materials.

Herein, we report our investigations into Li^+^ diffusion within LiFePO_4_, Nb- and V-doped LiFePO_4_. The two doped LiFePO_4_ samples both displayed enhanced cycling performance at high discharge rates in comparison with the undoped LiFePO_4_ sample^21,23^, and the µSR results allowed the unambiguous comparison of Li-ion mobility on a local level within these samples. This increased insight into Li^+^ diffusion processes present the future possibility of optimising doped compositions to give improved Li-ion battery performance.

## Methods

The synthesis of pure LiFePO_4_, Nb- and V-doped LiFePO_4_ materials have been described in detail in previous publications^21,23^. Briefly, these carbon-coated lithium iron phosphate samples (where the C is amorphous) were synthesized using a pilot-scale continuous hydrothermal flow synthesis (CHFS) reactor, described in detail in the Supporting Information and elsewhere^24^. These samples were heat-treated at 700 °C for 3 h (5 °C min^−1^ ramp rate) to graphitize the carbon coatings. Undoped LiFePO_4_ was selected for muon analysis as a control as well as the optimal performing dopant compositions of LiFe_0.99_Nb_0.01_PO_4_ and LiFe_0.95_V_0.05_PO_4_, which were named δLFP, δLFNP(1.0) and δLFVP(5), respectively (the δ term denotes these samples were heat-treated).

The µSR experiments were conducted at the ISIS pulsed muon and neutron source on the EMU instrument^25^. The data were analysed using the Windows Muon Data Analysis (WiMDA) program^26^. These samples were prepared for analysis by transferring *ca*. 1 g into Ti cavities with a Ti foil window. Ti was chosen as a sample holder material because it has negligible internal magnetic fields and therefore gave a simple background feature which could be easily subtracted in the analysis.

**Figure 1:**
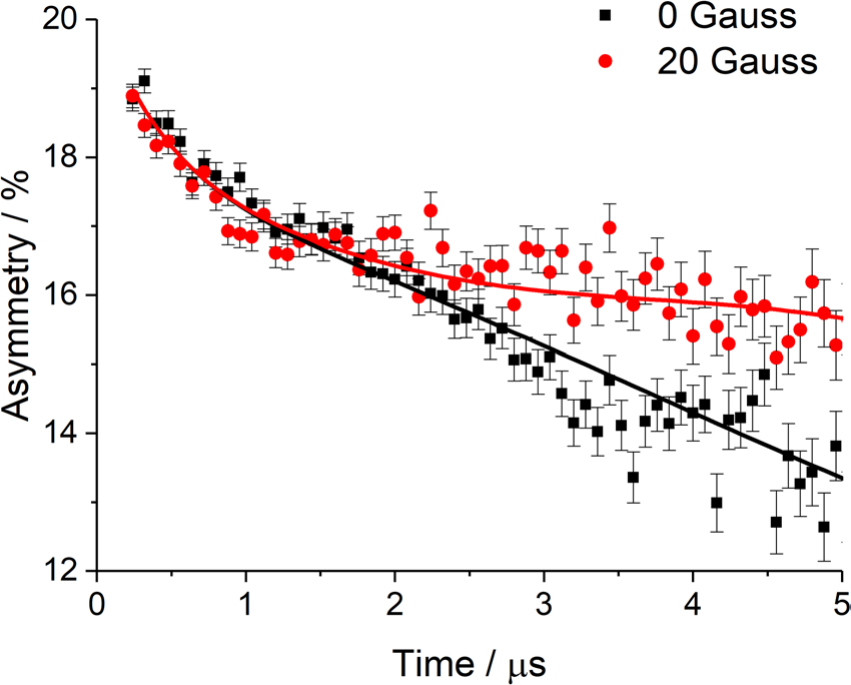
Representative muon decay asymmetry for sample δLFP, showing the raw data with the fit overlaid, as a function of time at various magnetic fields.

Spin-polarised positive muons were implanted into the δLFP, δLFNP(1.0) and δLFVP(5) samples, where they occupied interstitial sites for a mean lifetime of 2.2 µs before decaying. The muon spin direction was affected by the local magnetic field or diffusing species near the implantation site. The asymmetry in the count rate of the positrons, A(t), was measured in two arrays of detectors on opposite sides of the sample. While the implanted muons are almost 100% polarised, their three-body decay into a positron and two neutrinos, as well detector geometry constraints, limited the positron count rate asymmetry to *ca*. 25% on the EMU instrument. In order to probe the lithium diffusion behaviour in the three samples, measurements were in the temperature range 100 to 400 K for all samples. At each temperature, measurements were made at multiple magnetic fields (applied along the initial muon spin direction). These gave a way of comparing the applied field to the internal fields experienced by muons in the sample and constrained the model used for analysing the data more rigorously than could have been done with a single measurement. The Li^+^ diffusion was investigated in this study with µSR with zero applied field (ZF) and varying strengths of applied longitudinal field (LF) at 5, 10 and 20 G. Representative muon decay asymmetry spectra at 290 K for sample LFP at 0 and 20 G are shown in Fig. 1.

The spectra arose from a combination of a rapid interaction with the paramagnetic iron moments, and a slower interaction with the ^6^Li, ^7^Li and ^31^P nuclear magnetic moments. This allowed Li^+^ diffusion to be extracted in a similar manner to previous µSR studies of LiFePO_4_^7,8^. The data sets were fitted using four different parameters. Firstly, Keren’s analytic generalization of the Abragam function was applied, which has previously been altered to describe fluctuations due to Li^+^ or µ^+^ diffusion (assuming a Gaussian distribution of local fields)^7^. This function was chosen due to the increased relative speed of computation of the Keren function compared to the Kubo-Toyabe function used in previous studies^5^. Secondly, an exponential relaxing function was used, accounting for the rapid interaction with iron electronic magnetic moments. Thirdly, a baseline asymmetry was used to account for weak interactions with Ti and C present in the sample holder and sample, respectively. Finally, an additional exponentially decaying function was added as a separate term, to account for interactions with minor ferric impurities. These were not observed by XRD, so are assumed to be very minor, or amorphous. By fitting with these parameters, the muon fluctuation rate (*v*_Li_) due to Li^+^ diffusion and the local field distribution (Δ), could be extracted.

## Results and Discussion

**Figure 2:**
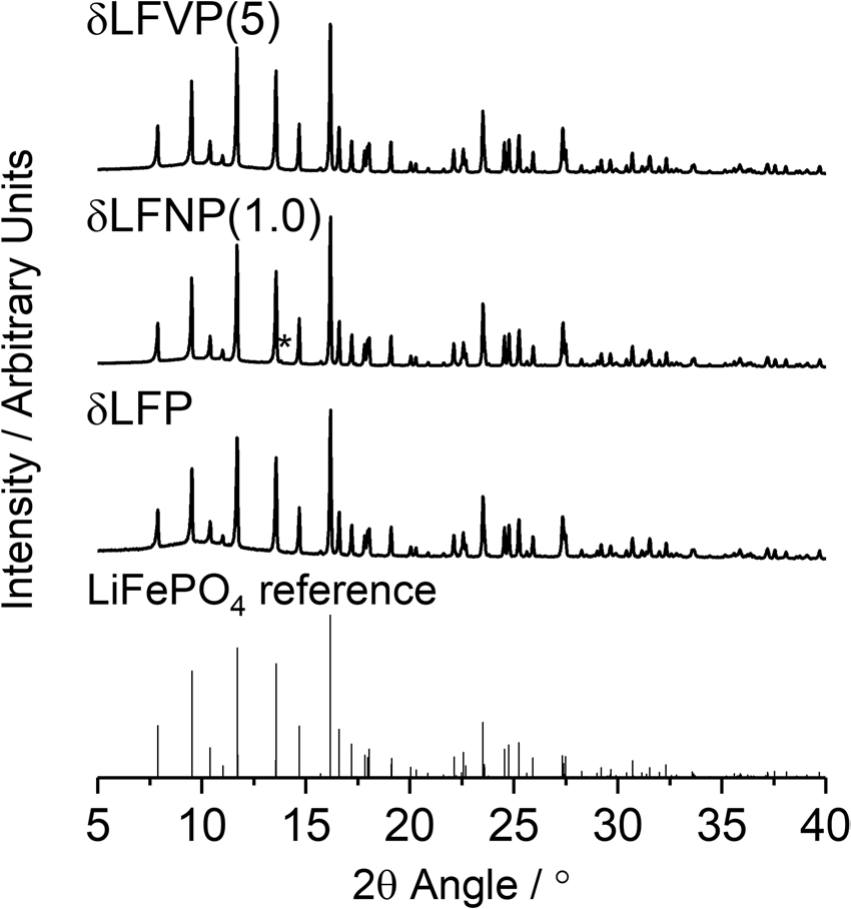
X-Ray diffraction patterns of δLFP, δLFNP(1.0) and δLFVP(5) with an LiFePO_4_ standard (PDF Card No. 01-070-6684), with the Fe_2_P_2_O_7_ minor impurity phase peak (PDF Card No. 00-076-1672, present in 1.5 vol% from Rietveld refinement) highlighted with an asterisk. A more detailed figure of the impurity phase peak is shown in Figure S2.

X-Ray diffraction analysis of the δLFP, δLFNP(1.0) and δLFVP(5) samples, confirmed each crystallised in the *Pnma* space group of the olivine structure (Fig. 2). The high-quality XRD revealed a minor impurity peak in δLFNP(1.0) (at 2θ~13.8°), which is consistent with Fe_2_P_2_O_7_ and has been observed previously in heat-treated carbon-coated olivines (Figure S2)^27^. The lattice parameters were extracted from Rietveld analysis using MAUD (Material Analysis Using Diffraction) software^28^, and are displayed in Table 1 and plots of the refinements are displayed in Figures S3–S5. The dopants had a minor effect on the lattice parameters, with a small contraction of the *b*-axis and lengthening of the *c*-axis, consistent with previous studies of doped samples^20,21^. This crystallographic change is primarily due to the different ionic radii of V^3+^ (0.64 Å) and Nb^5+^ (0.64 Å) occupying the Fe^2+^ (0.78 Å) site. Occupation of V on the Fe site with a Li vacancy as a charge-compensation mechanism in LiFePO_4_ was confirmed by the authors previously for δLFVP(5) with a combined Extended X-Ray Absorption Spectroscopy (EXAFS) and Density Functional Theory (DFT) study^21^, with the V:Fe ratio quantified as 5:95 by ICP-AES analysis. In addition, the authors confirmed an even dispersion of Nb within Nb-doped LiFePO_4_ samples^23^, with no Nb-containing impurity phases observed. The proportion of Nb within LiFePO_4_ was found to approximately match the stoichiometry of the precursors (Figures S6 and S7).

**Table 1:** The lattice parameters and goodness-of-fit parameters calculated from Rietveld refinement.

Sample	*a*/Å	*b*/Å	*c*/Å	*V*/Å^3^	R_wp_	χ^2^
δLFP	10.32407 (14)	6.00399 (9)	4.69447 (7)	290.990 (13)	4.48	1.34
δLFNP (1.0)	10.32252 (9)	6.00098 (6)	4.69633 (5)	290.915 (8)	3.79	2.20
δLFVP (5)	10.32345 (9)	6.00260 (6)	4.69687 (5)	291.054 (8)	3.84	2.21

**Figure 3:**
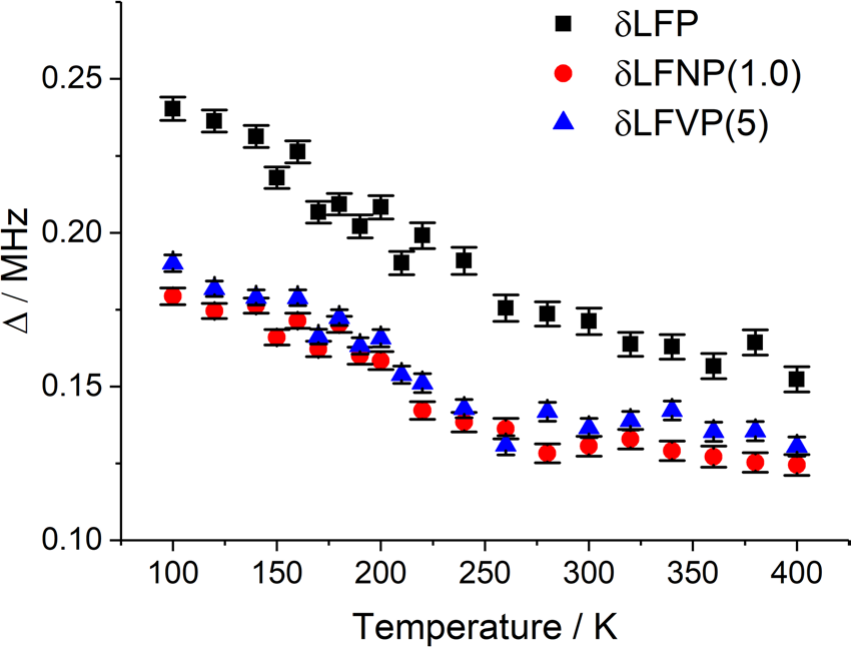
The local field distribution (with error bars) as a function of temperature for samples δLFP, δLFNP(1.0) and δLFVP(5).

The behaviour of Δ was similar to that reported previously by others for undoped LiFePO_4_, i.e. a steady decrease with increasing temperature, although the values for Δ were consistently lower for the doped samples (Fig. 3). The relative reduction in Δ seen in the doped samples herein cannot be definitively attributed, but could originate from an altered occupation of muon stopping sites, increased Li vacancies or changes in the muon mobility within the sample.

**Figure 4:**
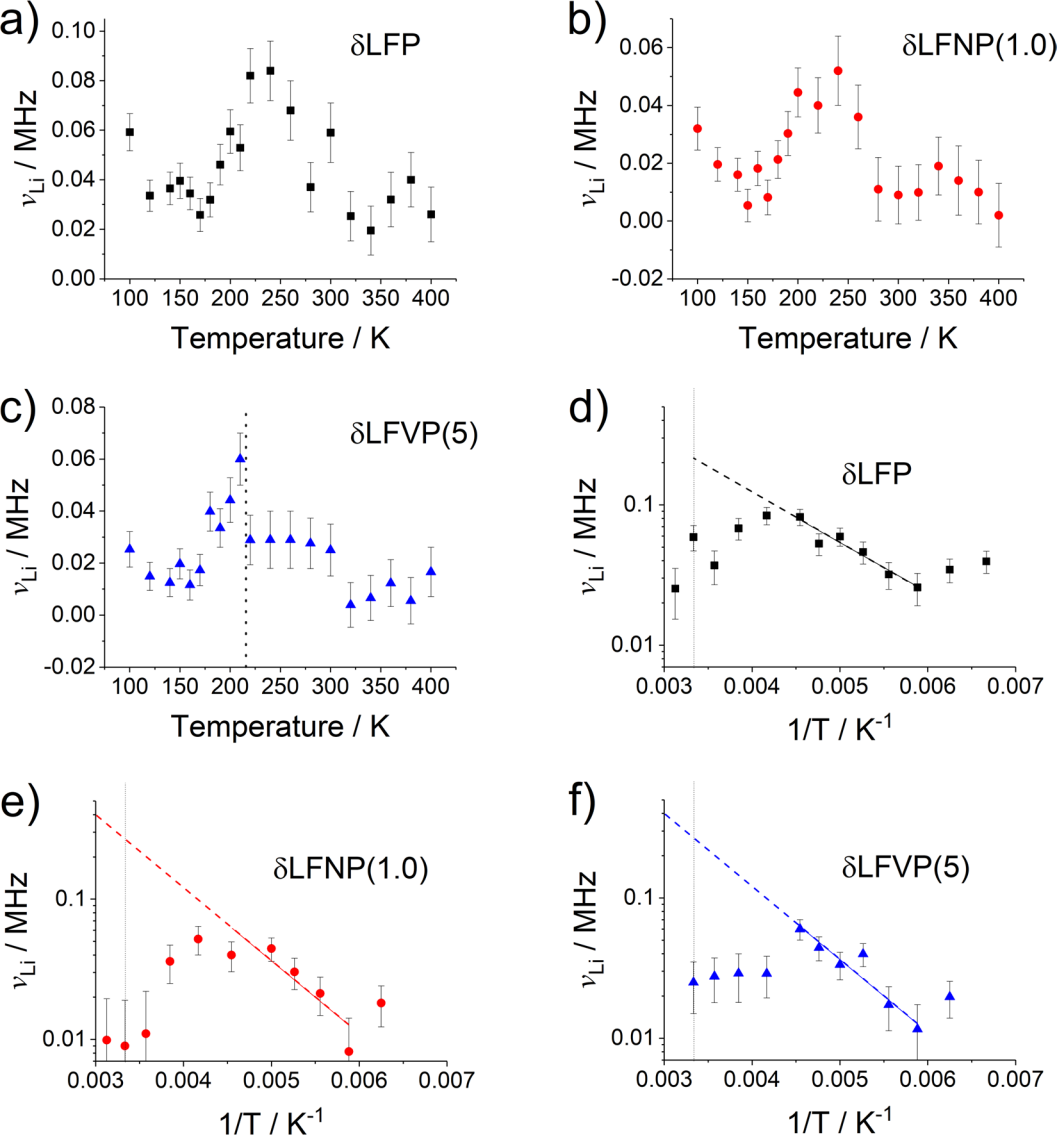
Plots of muon fluctuation rates *v*_Li_ vs Temperature for (**a**) δLFP, (**b**) δLFNP(1.0) and (**c**) δLFVP(5). Extrapolations of the muon fluctuation rate *v*_Li_ to room temperature on a log plot (indicated by the dotted line) vs inverse temperature (1/T) for (**d**) δLFP, (**e**) δLFNP(1.0) and (**f**) δLFVP(5).

All samples showed a characteristic increase and then decrease in *v*_Li_ with temperature, although the magnitude of *v*_Li_ was about 10% of that reported in the literature for undoped LiFePO_4_ samples previously (Fig. 4a–c)^6–8^. This may have been due to the significant embedding of muons in the carbon shell (range of 3 to 9 wt% carbon within the three samples, Table S1, corresponding to 1:2 and 1:1 molar ratios of C:LiFePO_4_), which would have detracted from the overall measured diffusion rate, but would not be expected to contribute to the observed fluctuation rate. For δLFP and δLFNP(1.0), an increase in *v*_Li_ with increasing T in the range *ca*. 180–250 K and a decrease thereafter was observed (Fig. 4a,b). In contrast, δLFVP(5) displayed a rapid increase of *v*_Li_ in the range 170–210 K, followed by a rapid decay above 210 K to a lower value of *v*_Li_ (0.03 MHz, Fig. 4c). This behaviour indicated there may be some observable difference in diffusion behaviour of δLFVP(5) and the other samples in this temperature range. However, given the relative error of the data points, further experiments are necessary to confirm the existence of any deviation from normal diffusion behaviour in vanadium-doped LiFePO_4_.

The hopping rate of Li^+^ can be converted to a diffusion coefficient according to Equation 1, where *N*_*i*_ is the number of Li sites in the *i*th path, *Z*_*v*,*i*_ is the vacancy fraction, and *s*_*i*_ is the hopping distance^29^. Therefore, as Li can diffuse in either direction along the 1D LiFePO_4_ diffusion tunnels, *n* = 2, where the number of vacant sites in each direction is 1, i.e. *N*_1_ = *N*_2_ = 1. For diffusion to occur, there must be a vacancy in the neighbouring sites, so *Z*_1_ = *Z*_2_ = 1. Therefore, Equation 1 simplifies to Equation 2. As Li^+^ diffusion in LiFePO_4_ has been shown to occur exclusively along the *b*-axis^30^, the hopping length is approximately *b*/2, and therefore D_Li_ can be estimated from *b*^2^*v*_Li_/4, and extrapolating *v*_Li_ against 1/T can determine a value for the Li-ion diffusion coefficient at room temperature (Fig. 4d–f). As the carbon coating contains no mobile lithium, it was assumed that the diffusion distance was accurately described by *b*/2.

1 $${{\rm{D}}}_{{\rm{Li}}}=\sum _{i=1}^{n}(\frac{1}{{N}_{i}}{Z}_{v,i}{s}_{i}^{2}{v}_{{\rm{Li}}})$$

2 $${{\rm{D}}}_{{\rm{Li}}}={s}_{i}^{2}{v}_{{\rm{Li}}}$$

Diffusion coefficients of approximately 2 × 10^−10^ cm^2^ s^−1^ were estimated for δLFP, δLFNP(1.0) and δLFVP(5), respectively, which were similar within error (Table 2). As a comparison, the diffusion coefficient values obtained here were consistent with those obtained by µSR for undoped LiFePO_4_ previously, pointing to the reliability of this technique for determining the diffusion properties of off-stoichiometry olivines^5,7,8^. The activation energies of Li^+^ diffusion (calculated from the gradient of diffusion coefficient against 1/T) were also consistent with previous analyses; E_a_ was in the range 70–100 meV for all samples^5,7,8^. For example, Baker *et al*. found D_Li_ values in the range 4–20 × 10^−10^ m^2^ s^−1^ and E_a_ values in the range 80–130 meV for the lithium-deficient olivines, Li_1−*x*_FePO_4_ (where 0 ≤ *x* ≤ 0.2)^7^. Indeed, Baker *et al*. found greater values of E_a_ for their Li-deficient samples, which suggests that Li deficiencies present in doped LiFePO_4_ (Table S2) may be responsible for any difference observed between samples in the temperature range 170–210 K. However, no quantitative difference in diffusion coefficient was observed between the undoped and doped samples when the low-temperature data was extrapolated to room temperature. This suggested that the intrinsic Li^+^ diffusion hopping rate at room temperature was not affected by doping, and that any enhancement of electrochemical performance observed must be due to other factors, such as increased electronic conductivity, or stabilisation of the Li_1−*x*_FePO_4_ and Li_*x*_FePO_4_ solid solutions. Such an observation could not be made via conventional techniques, such as impedance spectroscopy, as the two-phase delithiation mechanism of LiFePO_4_ prevents accurate probing of Li^+^ diffusion.

**Table 2:** The calculated diffusion coefficients and gradients from µSR.

Sample	D_Li_ @ 300 K/cm^2^ s^−1^	E_a_/meV
δLFP	1.8 ± 2 × 10^−10^	70 ± 10
δLFNP (1.0)	2.1 ± 20 × 10^−10^	100 ± 18
δLFVP (5)	2.3 ± 6 × 10^−10^	100 ± 30

## Conclusions

Li^+^ diffusion within V- and Nb-doped LiFePO_4_ samples, made *via* a continuous hydrothermal process^31^, have been characterised with muon spectroscopy for the first time. The calculated Li^+^ diffusion coefficients were close to the values previously reported for bulk and nanometric undoped LiFePO_4_. Thus, this report highlights the versatility of the μSR technique to analyse families of materials made by a variety of synthesis techniques. Within experimental error, the Li^+^ diffusion data suggested that electrochemical enhancements due to doping are not a result of improved local Li^+^ diffusion. Rather, the authors suggest that other factors, such as increased electronic conductivity or stabilisation of the Li_1−*x*_FePO_4_ and Li_*x*_FePO_4_ solid solutions may account for these enhancements. Therefore, these results have indicated the utility of μSR to provide key insights into the diffusive behaviour of doped LiFePO_4_, and could be applied to further battery materials in the future.

## Electronic supplementary material

Supplementary Information
