# Supplementary material for: Mechanistic insights of Li+ diffusion within doped LiFePO4 from Muon Spectroscopy
Source: Sci Rep. 2018 Mar 7;8:4114. doi: 10.1038/s41598-018-22435-1 (PMC5841297; doi:10.1038/s41598-018-22435-1)
Supplement: Supplementary file 1 — Supplementary Information [file 41598_2018_22435_MOESM1_ESM.pdf]

Mechanistic insights of Li^+ diffusion within doped $LiFePO_4$ from Muon Spectroscopy

Ian D. Johnson, Thomas E. Ashton, Ekaterina Blagovidova, Glen J. Smales, Mechthild Lübke, Peter J. Baker, Serena A. Corr and Jawwad A. Darr*

Supporting Information

Synthesis

Lithium iron phosphate samples with an amorphous carbon coating were synthesized using a pilot-scale continuous hydrothermal flow synthesis (CHFS) reactor incorporating a confined jet mixer (CJM), where schematics of both are shown in Figure S1a and Figure S1b respectively. Two aqueous precursor solutions were prepared. The first solution consisted of $FeSO_4 \cdot 7H_2O$ (99+%, Alfa Aesar, Heysham, UK), $VOSO_4 \cdot 5H_2O$ (17-23% V, Acros Organics, Loughborough, UK), $C_4H_4NNbO_9 \cdot 5H_2O$ (99.99%, Sigma-Aldrich Ltd., Dorset, UK), 0.375 M H_3PO_4 (85-88% wt%, Sigma Aldrich, Steinheim, Germany) and fructose (99%, Alfa Aesar, Heysham, UK) in deionized (D.I.) water. The first solution was varied as described in Table S1, where the sum of [Fe], [V] and [Nb] was 0.25 M and the concentration of fructose was 0.65 – 0.75 M. The second solution contained 0.8625 M $LiOH \cdot H_2O$ (99+%, Fischer Scientific, Loughborough, UK) in D.I. water. Each solution was pumped (200 mL min^{-1} flow rate each) into a T-piece mixer (0.25" internal diameter). The combined mixture of solutions 1 and 2 (400 mL min^{-1}) was pumped into the side arms of the CJM, where it rapidly combined with 400 mL min^{-1} (referred to 24.1 MPa and 20 °C) of supercritical water at 450 °C and 24.1 MPa (Figure S1b). The nanoparticles of $LiFePO_4$/C (either pure, doped with V or doped with Nb) rapidly formed in the mixture at a temperature of *ca.* 335 °C. The mixture flowed through the outlet pipe with an approximate residence time of 6.5 s and was then cooled to below 60°C through a pipe-in-pipe countercurrent heat exchanger. The cooled slurry flowed through a back-pressure regulator (BPR, Swagelok KHB series) which maintained the system pressure at 24.1 MPa, after which it was collected in a plastic container open to the atmosphere. The slurry was allowed to settle out and the supernatant (containing unreacted precursors and by-products) siphoned off. The concentrated slurry was further concentrated using a centrifuge (4500 r.p.m, 10 minutes), and repeatedly redispersed in D.I. water to clean the product. The cleaned slurry was placed in a freeze-drier (Virtis Genesis 35XL) and slowly heated from −60 °C to 25 °C, over 24 h under vacuum of < 13.3 Pa. The freeze-dried powder was subsequently heat-treated from ambient temperature up to 700 °C and held for 3 hours, with a heating rate of 5 °C min^{-1} under a flow of argon. The V-doped $LiFePO_4$ sample was extremely granular, and required gentle milling using a Retsch planetary ball mill PM-200 using a 1:1 w/w ratio of LFP and N-methyl pyrrolidone (NMP) with 4 mm zirconia balls at 400 rpm for 30 minutes to reduce the size of fused agglomerates.

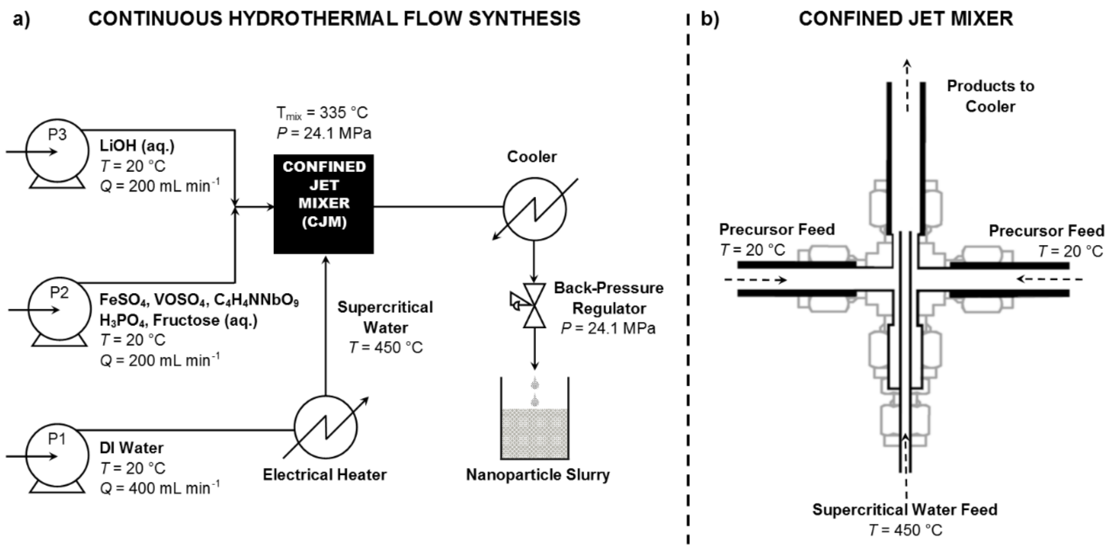

Figure S1 – a) a schematic of the Continuous Hydrothermal Flow Synthesis (CHFS) process; b) a schematic of the Confined Jet Mixer (CJM).

Table S1. The metal and fructose precursor concentrations used to produce samples δLFP, δLFNP(1.0) and δLFVP(5), and the proportion of carbon in the heat-treated samples.

Sample Name	$FeSO_4 \cdot 7H_2O$ / mM	$VOSO_4 \cdot 5H_2O$ / mM	$C_4H_4NNbO_9 \cdot 5H_2O$ / mM	Fructose / M	Carbon coating / wt%
δLFP	250	0	0	0.75	9.1
δLFNP(1.0)	247.5	0	2.5	0.65	2.9
δLFVP(5)	237.5	12.5	0	0.65	8.3

Materials Characterisation

High-quality XRD patterns of the samples were obtained on a STOE StadiP diffractometer in a 0.3 mm borosilicate glass capillary using Mo-Kα radiation (λ = 0.71 Å) over the 2θ range 5 – 60° with a step size of 0.5° and step time of 87 or 100 s. The wt% of carbon in the samples was determined using CHN analysis.

Energy-dispersive spectroscopy (EDS) was conducted using a Jeol JEM-1010 fitted with an Oxford Instruments XMaxN 80-T Silicon Drift Detector (SDD)) and processed using AZtec® software.

Chemical analysis for Li, Fe, V and P was performed by ICP-AES using dilute solutions of the samples dissolved in 1% HNO_3 (aq.) (Department of Earth Sciences, University College London).

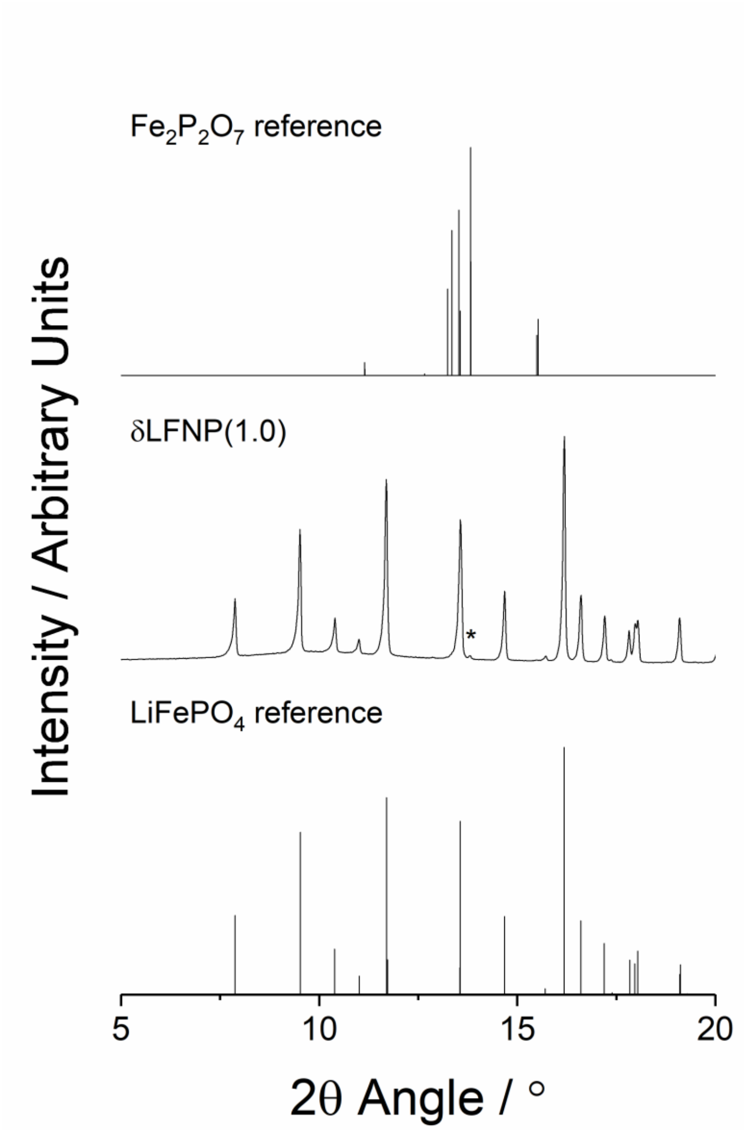

Figure S2. X-Ray diffraction of the δLFNP(1.0) sample showing a good match to $LiFePO_4$ (PDF Card No. 01-070-6684). The impurity peak at 13.8° 2θ is highlighted, and the suggested impurity phase $Fe_2P_2O_7$ included as a reference (PDF Card No. 01-076-1762).

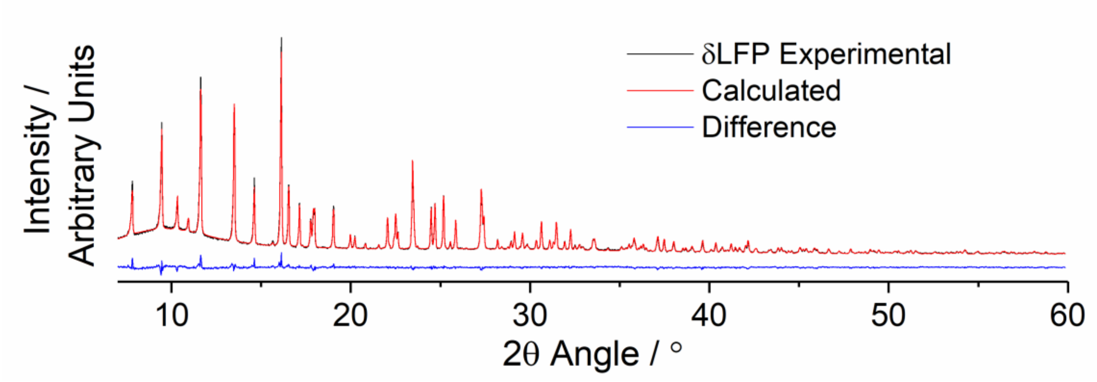

Figure S3. Experimental and calculated diffraction patterns for sample δLFP using Rietveld analysis. R_{wp} = 4.48, χ^2 = 1.34.

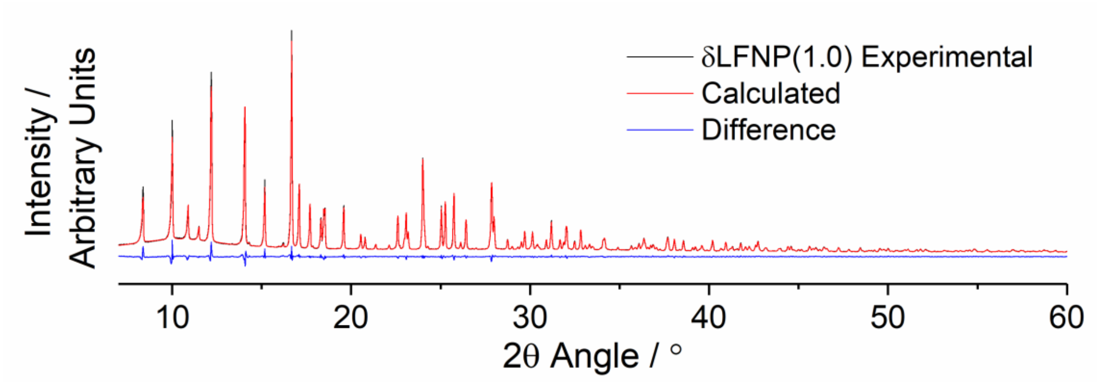

Figure S4. Experimental and calculated diffraction patterns for sample δLFNP(1.0) using Rietveld analysis. R_{wp} = 3.79, χ^2 = 2.20.

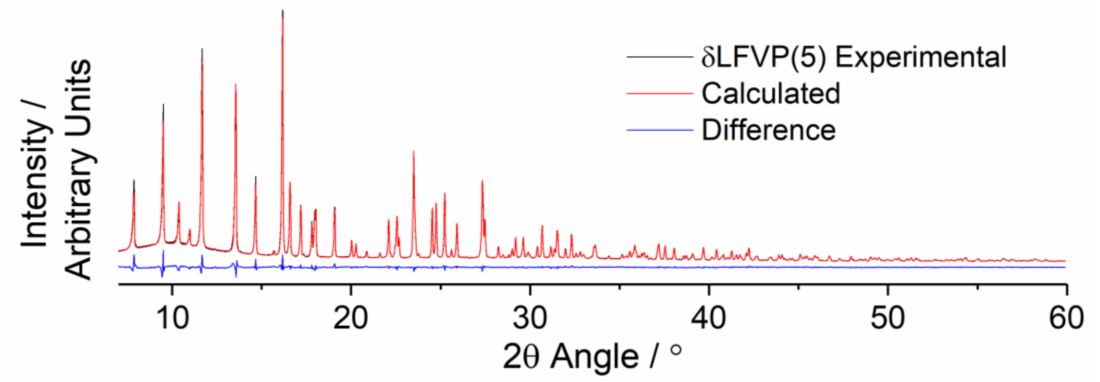

Figure S5. Experimental and calculated diffraction patterns for sample δLFVP(5) using Rietveld analysis. R_{wp} = 3.84, χ^2 = 2.17.

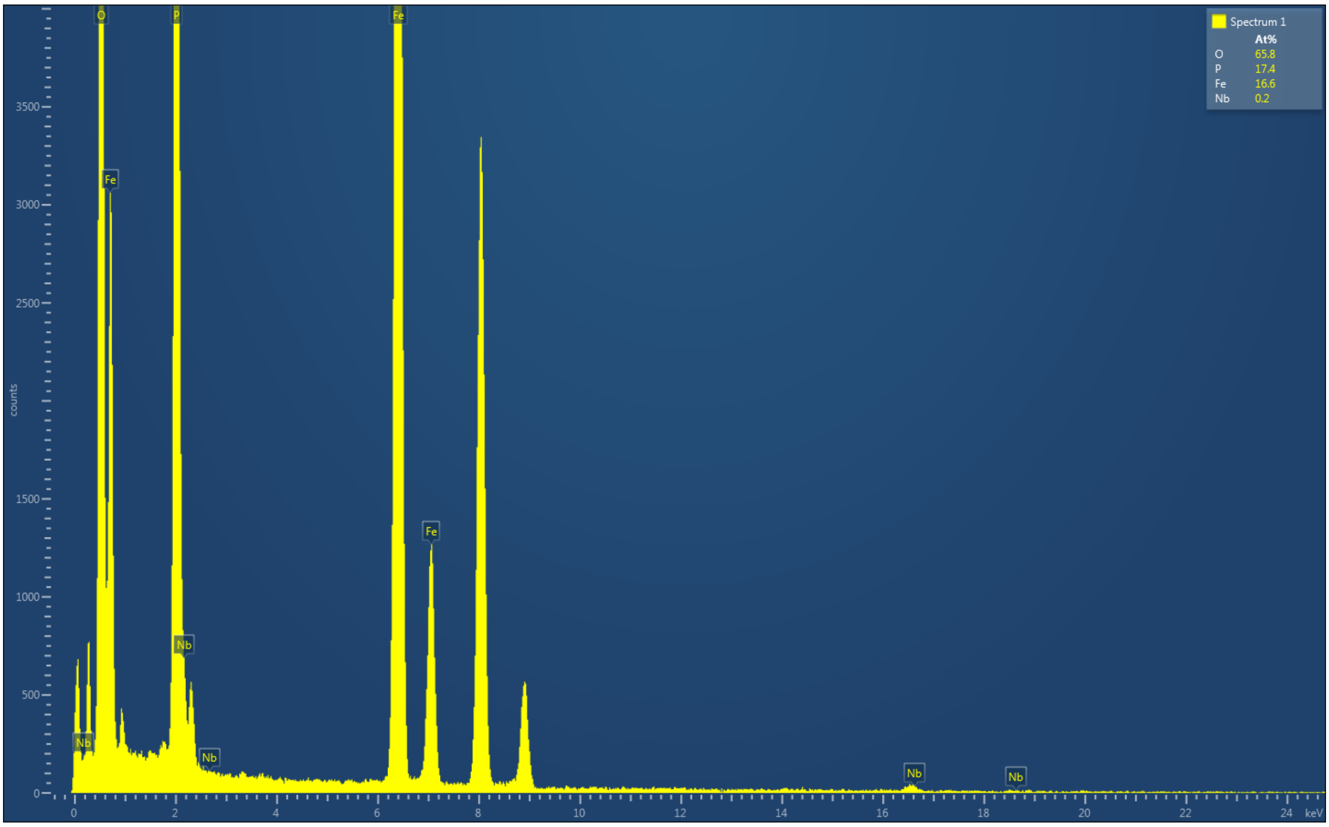

Figure S6. The EDS spectrum of ΔLFNP(1.5) from reference 25, with an approximate atomic ratio of Nb:Fe 1.2:98.8.

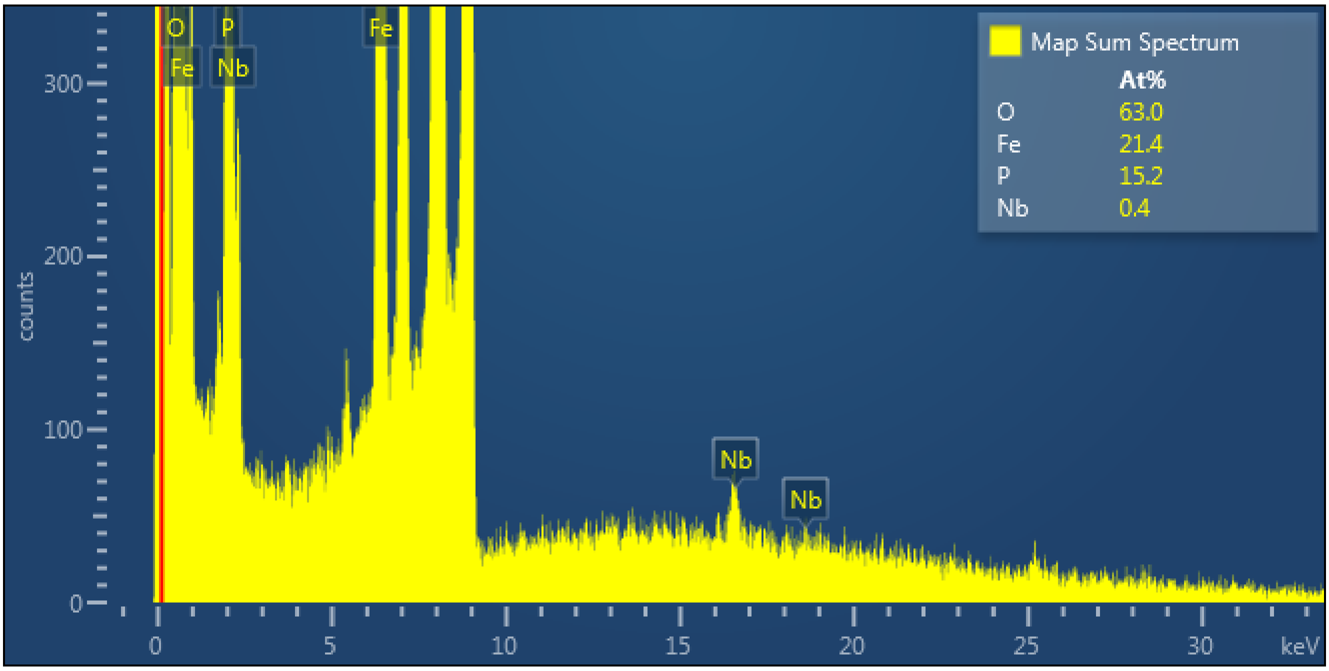

Figure S7. The EDS spectrum of ΔLFNP(2.0) from reference 25, with an approximate atomic ratio of Nb:Fe 1.8:98.2.

Table S2. The ratio of elements found by ICP-AES analysis in the doped and undoped $LiFePO_4$ samples, normalised to phosphorous. Nb did not produce a stable signal in the plasma, and could not be quantified.

Sample	Li/P	Fe/P	V/P	P/P
δLFP	1.05	1.04	0.00	1.00
δLFNP(1.0)	1.013	1.013	0.00	1.00
δLFVP(5)	0.97	0.96	0.05	1.00
